# RNA-Seq Analysis of Glycolysis Regulation of Avian Leukosis Virus Subgroup J Replication

**DOI:** 10.3390/ani14172500

**Published:** 2024-08-28

**Authors:** Ting Yang, Lingling Qiu, Shihao Chen, Zhixiu Wang, Yong Jiang, Hao Bai, Yulin Bi, Guobin Chang

**Affiliations:** 1 College of Animal Science and Technology, Yangzhou University, Yangzhou 225009, China; 2 Key Laboratory for Animal Genetics & Molecular Breeding of Jiangsu Province, Yangzhou University, Yangzhou 225009, China; 3 Institutes of Agricultural Science and Technology Development, Yangzhou University, Yangzhou 225009, China; 4 Joint International Research Laboratory of Agriculture and Agri-Product Safety of Ministry of Education of China, Yangzhou University, Yangzhou 225009, China

**Keywords:** glycolysis, ALV-J, CircRNA, virus replication

## Abstract

**Simple Summary:**

Avian Leukosis virus (ALV) is a virus that is widespread in poultry and hinders the healthy development of the poultry industry. In this study, we found that inhibition of DF1 cell glycolysis can reduce the replication of ALV-J virus. Next, we performed RNA-seq on ALV-J-infected and ALV-J-infected cells treated with glycolysis inhibition. Then, we analyzed the function of source and target genes of different expressed circular RNAs (DE circRNAs) and constructed a circRNA–miRNA–gene axis. The results of this study reveal the key role of several circRNAs in the replication of the ALV-J virus and lay a foundation for understanding the molecular mechanism of ALV-J virus replication regulation.

**Abstract:**

Avian Leukosis virus (ALV) is a widely spread virus that causes major economic losses to the global poultry industry. This study aims to investigate the effect of glycolysis on the replication of the ALV-J virus and identify the key circular RNAs that regulate the replication of the ALV-J virus. We found that glucose uptake, pyruvate content, and lactate content in DF1 cells were increased after ALV-J infection. Moreover, inhibiting the glycolysis of ALV-J-infected DF1 cells reduced the replication of the ALV-J virus. To further study the mechanism of glycolysis in the replication of the ALV-J virus, we performed RNA-seq on ALV-J-infected and ALV-J-infected cells treated with glycolysis inhibition. RNA-seq results show that a total of 10,375 circular RNAs (circRNAs) were identified, of which the main types were exonic circular RNAs, and 28 circRNAs were differentially expressed between ALV-J-infected and ALV-J-infected cells treated with glycolysis inhibition. Then, we performed functional enrichment analysis of differentially expressed circRNA source and target genes. Functional enrichment analysis indicated that some circRNAs might be involved in regulating the replication of the ALV-J virus by influencing some pathways like glycolysis/gluconeogenesis, the NOD-like receptor signaling pathway, MAPK signaling pathway, p53 signaling pathway, Toll-like receptor signaling pathway, Insulin signaling pathway, and Apoptosis. This study revealed the effect of glycolysis on the replication of the ALV-J virus in DF1 cells and its possible regulatory mechanism, which provided a basis for understanding the factors influencing the replication of the ALV-J virus and reducing the rate of infection of the ALV-J virus in poultry.

## 1. Introduction

Avian Leukosis virus (ALV) is an avian retrovirus that causes tumor disease, immunosuppression, and other poultry production-related problems, and has long caused significant economic losses in the global poultry industry [1]. To date, there are 10 subpopulations of ALV, of which the A, B, C, D, E, J, and K subpopulations have been isolated from chickens, each of which has a different function [2,3,4]. The properties of these subgroups depend on the viral envelope glycoproteins, which determine the serum neutralization properties of the virus, the pattern of viral interference, and the range of viral infection [5]. Since the isolation of ALV-J in China in 1999, its genome has changed significantly, its host range has expanded, and its transmission ability and pathogenicity have increased, which has brought serious harm to broilers, laying hens, and local breeds in China, not only causing huge economic losses for the poultry industry but also seriously threatening the provenological safety of domestic poultry [6]. To reduce the impact of the ALV-J virus on broiler production, researchers have taken a series of measures to reduce the transmission of the ALV-J virus; however, the J subgroup-related virus still brings many adverse effects on the healthy development of the poultry industry [7].

Viral infection can lead to changes in metabolic pathways in host cells, including glycolysis, amino acid, carbon metabolic, and nucleotide synthesis. The survival and pathogenesis of both carcinogenic and non-carcinogenic viruses that infect host cells depend on intracellular glycolysis [8,9]. Viruses need energy to survive and replicate after infecting cells, and this energy comes from the host cell’s glycolysis and other metabolic pathways; so, inhibition of glycolysis can prevent many viruses from replicating [8]. Studies have shown that inhibiting glycolysis with glycolytic inhibitors such as 2DG and Oxamate could reduce viral RNA synthesis of Norovirus and Dengue virus [10,11]. Therefore, glycolysis is necessary for many aspects of viral replication.

Circular RNA (CircRNA), an RNA molecule with a circular structure, was initially considered useless, but its importance has been increasingly recognized as researchers have discovered that it has important and complex functions [12]. Circular RNAs can be divided into three types based on their composition, including exonic circular RNAs (ecircRNAs), circular intronic RNAs, and exon–intron circular RNAs (EIciRNAs) [13]. Numerous studies have shown that circRNAs have many significant characteristics, including multiplicity, abundant expression, stability, and conservation, and these characteristics suggest that circRNAs have a variety of potential biological functions [14,15,16,17,18]. In tumor cells, circRNAs can influence glycolytic pathways. Studies have shown that knocking out the circCCDC66 gene in PTC cells can significantly inhibit glucose metabolism [19]. Similar studies have shown that inhibiting hsa_circ_0011290 in PTC can inhibit glucose uptake, reduce lactic acid production, increase ATP content, and inhibit cell proliferation and cell viability [20]. In addition, circular RNAs have been reported to participate in host–virus interactions. Studies have shown that circRNA can participate in cellular responses to herpes simplex virus 1, human cytomegalovirus, and HIV infection through the circRNA–miRNA–gene regulatory axis [21,22,23]. In a previous study, we also found that circ_PIAS1 can regulate the apoptosis of DF1 cells and participate in ALV-J infection [24]. These studies suggest that circRNA plays an important role in the glycolytic pathway and the viral replication process.

In this study, we aimed to investigate the effects of glycolysis on viral replication after ALV-J infection in DF1 cells. The results showed that glycolysis could affect the replication of ALV-J by regulating the expression level of circRNAs. The results provided the basis for reducing the infection of ALV-J in the poultry industry.

## 2. Materials and Methods

### 2.1. Cell Culture

DF1 cells were purchased from the American Type Culture Collection (ATCC) and all cells were cultured in DMEM and added to 10% FBS. Cell culture conditions were 37 °C and 5% CO_2_.

### 2.2. ALV-J-Infected Treatment

DF1 cells were seeded in a 6-well cell culture plate. ALV-J virus was inoculated into DF1 cells at a multiplicity of infection (MOI) of five after the confluency of the cell monolayer reached 60%. After 2 h, replace the medium with a new medium with 1% FBS content. DF1 cells were continued to be cultured for 48 h, and subsequent experiments were performed. ALV-J JS09GY3 strain was acquired from Professor Aijian Qin of Yangzhou University.

### 2.3. 2-Deoxy-d-Glucose Treatment

DF1 cells were seeded in a 6-well cell culture plate. The 2-Deoxy-d-glucose (2-DG, a glycolytic inhibitor) (Beyotime, Shanghai, China) was added to the medium after the confluency of the cell monolayer reached 60%. DF1 cells continued to be cultured for 48 h, and subsequent experiments were performed.

### 2.4. Glucose Uptake Assay

DF1 cells were seeded in a 6-well cell culture plate and the ALV-J virus (MOI 5)/2-DG was added to the medium after the confluency of the cell monolayer reached 60%. Then, the DMEM culture medium was replaced by a glucose-free medium with 10% FBS. After incubating the cells for another 30 min, a fluorescent glucose analog 2-(N-(7-nitrobenzo-2-oxazo-1,3-diazole-4-amino)-2-deoxyglucose(2-NBDG) (MCE, HY-116215, Shanghai, China) was added at a final concentration of 100 μM for 15 min and cells were collected and washed twice with PBS (Solarbio, Beijing, China). Finally, a Multimode micropore detection system (EnSpire, PerkinElmer, Waltham, MA, USA) was used to measure the OD value of DF1 cells at 488 nm.

### 2.5. Pyruvate Content Assay

First, DF1 cells were seeded in a 6-well cell culture plate. Then, the ALV-J virus (MOI 5)/2-DG was added to the medium after the confluency of the cell monolayer reached 60%. A Pyruvate content assay kit (Jiancheng Bioengineering, Nanjing, China) was used to detect pyruvate content according to the instructions. Finally, a Multimode micropore detection system (EnSpire, PerkinElmer, Waltham, MA, USA) was used to measure the OD value of DF1 cells at 505 nm.

### 2.6. Lactate Content Assay

First, DF1 cells were seeded in a 6-well cell culture plate. Then, the ALV-J virus (MOI 5)/2-DG was added to the medium after the confluency of the cell monolayer reached 60%. A Lactic acid (LD) content assay kit (Jiancheng Bioengineering, Nanjing, China) was used to detect lactate content according to the instructions. Finally, a Multimode micropore detection system (EnSpire, PerkinElmer, Waltham, MA, USA) was used to measure the OD value of DF1 cells at 530 nm.

### 2.7. Total RNA Extraction, cDNA Synthesis, and Real-Time Quantitative PCR (qRT-PCR)

DF1 cells were seeded in a 12-well cell culture plate. ALV-J were inoculated into DF1 cells at a multiplicity of infection (MOI) of five after the confluency of the cell monolayer reached 60%. After 2 h, replace the medium with a new medium with 1% FBS content. DF1 cells were collected at 12 h, 24 h, 36 h, and 48 h. Then, the TRIzol Reagent (Takara, Dalian, China) was used for the total RNA extraction. HiScript III RT SuperMix for qPCR (+gDNA Wiper) (Vazyme, Nanjing, China) was used for cDNA synthesis, and the ChamQ Universal SYBR qPCR Master Mix (Vazyme, Nanjing, China) was used to detect gene expression levels. The QuantStudio 5 real-time PCR instrument was used for qRT-PCR. Primers were designed with Primer 5.0, and synthesized by Tsingke Biotech (Nanjing, China). The sequence of these primers is listed in Table 1. The chicken β-actin gene was used as the housekeeping gene. The expression levels of all genes were measured using the 2^−∆∆Ct^ method [25].

### 2.8. CCK-8 Assay

First, DF1 cells were seeded in a 96-well cell culture plate. Then, the 2-DG was added to the medium after the confluency of the cell monolayer reached 30%. After DF1 cells were treated for 48 h, cell activity was detected using a CCK-8 assay kit (Vazyme, Nanjing, China) in strict accordance with the instructions. Finally, a Multimode micropore detection system (EnSpire, Perkin Elmer, Waltham, MA, USA) was used to measure the OD value of DF1 cells at 450 nm.

### 2.9. Western Blot Assay

First, DF1 cells were seeded in a 6-well cell culture plate. Second, the ALV-J virus was inoculated into DF1 cells at a multiplicity of infection (MOI) of five after the confluency of the cell monolayer reached 60%. Two hours later, a 2-DG inhibitor was added. After DF1 cells were treated for 48 h, the protein was extracted using a RIPA lysate (Beyotime, Shanghai, China), and the protein concentration was detected using a BCA kit (Beyotime, Shanghai, China). Then, we used a 12% sodium dodecyl sulfate-polyacrylamide (SDS) gel (GenScript, Nanjing, China) to separate target proteins and target proteins were transferred onto a polyvinylidene difluoride (PVDF) membrane (Solarbio, Beijing, China). Finally, we incubated PVDF membranes with primary and secondary antibodies, respectively. An enhanced chemiluminescence solution is used for protein blot display (Beyotime, Shanghai, China). The primary antibodies included Mouse Anti-gp85, JE9 (Professor Aijian Qin of Yangzhou University), and Rabbit Anti-β-Actin (Novus Biologicals, Littleton, CO, USA). The secondary antibodies included HRP Goat Anti-Mouse IgG(H+L) (ABclonal, Wuhan, China) and HRP Goat Anti-Rabbit IgG(H+L) (ABclonal, Wuhan, China).

### 2.10. RNA Extraction, Library Construction, Sequencing, and Data Quality Control

Total RNA was extracted from DF1 cell samples using a Trizol reagent (Invitrogen, Carlsbad, CA, USA). RNA concentration was detected at OD 260/280 using a Nanodrop ND-2000 ultra-micro spectrophotometer (Thermo Fisher Scientific, Waltham, MA, USA). RNA quality was detected by an Agilent 2100 Bioanalyzer (Agilent Technologies, Palo Alto, CA, USA). RNA libraries were constructed using a TruSeq RNA-Seq Library Prep Kit v.2 (Illumina, Shanghai, China). Then, the RNA libraries were sequenced on the Illumina Novaseq^TM^ 6000 sequence platform by LC Bio-Technology Co., Ltd. (Hangzhou, China). Subsequently, for quality control, we used the FastQC (0.10.1) to acquire high-quality clean reads, and these clean reads were compared with a ribosomal database using the Bowtie2 [26]. The reads were aligned to the reference genome using Tophat 2 (2.0.4) and tophat-fusion (2.1.0) after being compared with a ribosomal database [27].

### 2.11. Identification and Differential Expression Analysis of circRNAs

CIRCExplorer2 (2.2.6) and CIRI (2.0.2) were used to de novo assemble the mapped reads to circular RNAs at first. Then, back-spliced junction reads were identified in unmapped reads by tophat-fusion and CIRCExplorer2 or CIRI. All samples were generated unique circular RNAs. The types, expression, and chromosomal distributions of these identified circRNAs were determined by statistical analyses. Subsequently, the identified circRNAs were annotated using BLAST searches according to the circBass database [28]. The differentially expressed circRNAs (DE circRNAs) across samples or groups were identified using the edgeR package (3.22.5). Among the ALV-J and ALV-J + 2-DG groups, the circRNAs with the parameter of *p*-value < 0.05 (*p*-value was corrected using the Benjamini and Hochberg method) and a fold change > 2 or fold change < 0.5 were considered differentially expressed circRNAs.

### 2.12. Target Gene Prediction and Functional Enrichment Analysis of Differential circRNAs

After the identified circRNAs were annotated using the circBass database, the differentially expressed circRNAs were predicted as miRNA targets using TargetScan (5.0) and miRanda (3.3a) with TargetScan_score ≥ 50 and miranda_Energy < −10. MiRTarBase (6.1) and TarBase (9.0) were used to predict messenger RNAs (mRNAs) [29,30]. The source and target genes of the DE circRNAs were analyzed for Gene Ontology (GO) and Kyoto Encyclopedia of Genes and Genomes (KEGG) functional enrichment [31,32].

### 2.13. Validation of DE circRNAs

To ensure the reliability of RNA-seq results, qRT-PCR was performed to verify the expression patterns of DE circRNAs between the two groups. Eight DE circRNAs were randomly chosen, and primers of these circRNAs were designed. The sequence of these primers is listed in Table 2. The chicken β-actin gene was used as the housekeeping gene. The expression levels of the circRNAs were measured using the 2^−∆∆Ct^ method [25].

### 2.14. Statistical Analysis

Statistical analysis was performed using the SPSS 25.0 software (SPSS Inc., Chicago, IL, USA) and the analysis of two-group comparisons using one-way ANOVA. *p* < 0.05 (*), *p* < 0.01 (**), or *p* < 0.001 (***) represent statistically significant, and all data are shown as means ± SD (standard deviation).

## 3. Results

### 3.1. ALV-J Elevates Glucose Uptake and Glycolysis in DF1 Cells

**Figure 1:**
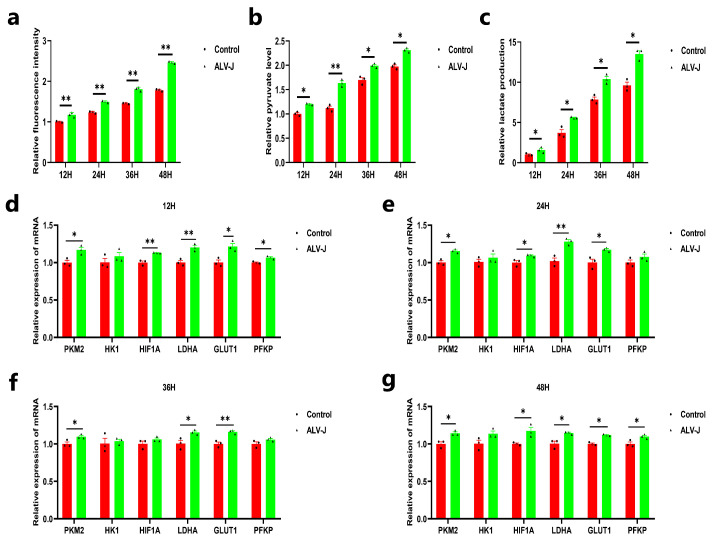
ALV-J elevates glucose uptake and glycolysis in DF1 cells. (**a**) The glucose uptake ability after DF1 cells were infected with the ALV-J virus. (**b**) The contents of pyruvate after DF1 cells were infected with the ALV-J virus. (**c**) The contents of lactate after DF1 cells were infected with the ALV-J virus. (**d**–**g**) The mRNA expression level of glycolysis-related genes (PKM2, HK1, HIF1A, LDHA, GLUT1, PFKP) after DF1 cells were infected with the ALV-J virus. *p* < 0.05 (*) or *p* < 0.01 (**) represent statistically significant, and all data are shown as means ± SD (standard deviation).

To understand whether ALV-J infection affects glucose metabolism, we infected DF1 cells with ALV-J to monitor glucose uptake ability using 2-NBDG following ALV-J infection. The results showed that ALV-J-infected cells significantly increased glucose uptake compared to uninfected cells at 12, 24, 36, and 48 h post-infection (Figure 1a). Then, we detected pyruvate and lactate production. The results showed that ALV-J-infected cells produced more pyruvate and lactate than uninfected cells (Figure 1b,c). In addition, we also examined the mRNA expression level of glycolysis-related genes. Similarly, ALV-J infection significantly increased the mRNA expression of glycolysis-related genes, such as glycolytic enzymes and glucose transporters (Figure 1d–g). These findings suggested that ALV-J promotes glucose uptake and glycolysis in infectious cells.

### 3.2. Inhibition of DF1 Cell Glycolysis Reduces ALV-J Replication

**Figure 2:**
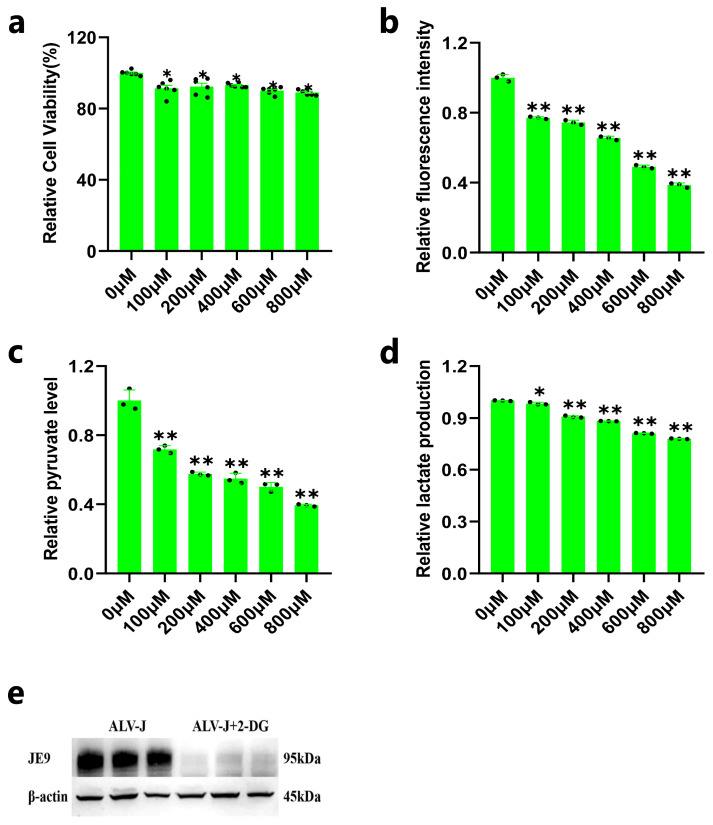
Inhibition of DF1 cell glycolysis reduces ALV-J replication. (**a**) The cell viability after exposing DF1 cells to different concentrations of 2-DG. (**b**) The glucose uptake ability after exposing DF1 cells to different concentrations of 2-DG. (**c**) The contents of pyruvate after exposing DF1 cells to different concentrations of 2-DG. (**d**) The contents of lactate after exposing DF1 cells to different concentrations of 2-DG. (**e**) The protein expression of JE9 after ALV-J-infected DF1 cells was treated with a 100 µM concentration of 2-DG. *p* < 0.05 (*) or *p* < 0.01 (**) represent statistically significant, and all data are shown as means ± SD (standard deviation).

We further explored whether glycolysis could affect the replication of ALV-J in DF1 cells. To confirm the optimal concentration of 2-DG for culturing DF1 cells, we detected the cell viability by exposing DF1 cells to different concentrations of 2-DG. The results showed that DF1 cell viability decreased to some extent after culture with various concentrations of 2-DG (Figure 2a). Additionally, the glucose uptake and the contents of pyruvate and lactate in DF1 cells decreased significantly after inhibiting the glycolysis of DF1 cells with 2-DG (Figure 2b–d). Based on these results, we treated ALV-J-infected DF1 cells with glycolytic inhibition (100 µM concentration of 2-DG) and detected the expression of the JE9 protein. The result showed that the protein level of JE9 was significantly decreased after ALV-J-infected DF1 cells were treated with a 100 µM concentration of 2-DG (Figure 2e), which indicated that inhibition of DF1 cell glycolysis could reduce ALV-J replication.

### 3.3. Overview of RNA Sequencing Data and Identification of Circular RNAs

**Figure 3:**
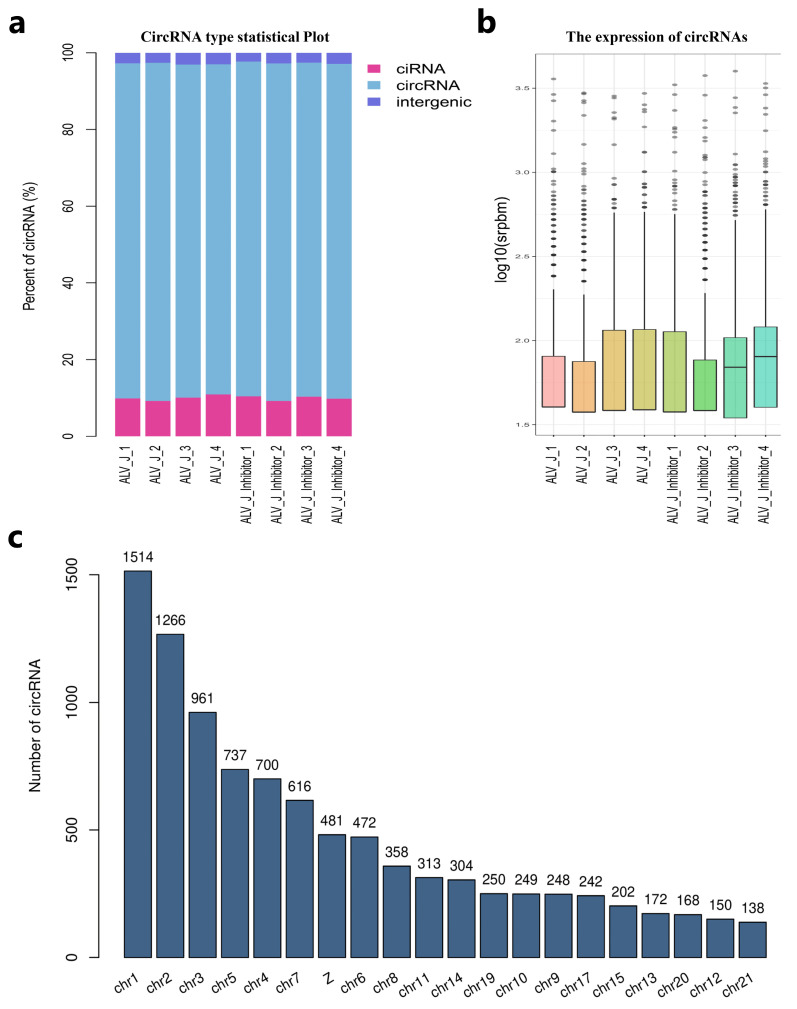
Overview of RNA sequencing data and identification of circular RNA. (**a**) Percent of identified circRNAs in various types. (**b**) The circRNA expression distribution for each sample. (**c**) The chromosomal location of the circRNAs.

To understand the possible mechanism of glycolysis affecting the replication of the ALV-J virus, we performed genome-wide circRNA-seq to identify the circRNAs affected by glycolysis in ALV-J-infected DF1 cells. We acquired 567,816,378 reads sequenced from all eight samples (Supplementary Table S1). After filtering the low-quality reads and adaptors, 505,812,710 clean reads were mapped to the chicken genome (Supplementary Table S1). Additionally, the unique mapped reads, multi-mapped reads, pair-end mapped reads, reads mapped to sense strands, reads mapped to antisense strands, non-splice reads, splice reads, unmapped reads, and back-spliced junctions reads are also analyzed (Supplementary Table S1). Then, we found 11,066 circular RNAs generated by 7588 genes among 1,396,846 candidate back-spliced junction reads (Supplementary Table S2). There are three types of circular RNA among the eight samples, mainly circular RNA, with a total of 8516. This was followed by ciRNA and intergenic circular RNA, with 1623 and 236 types, respectively (Figure 3a). The srpbm box graph of samples showed the circRNA expression distribution (Figure 3b). All these circular RNAs were widely distributed on chicken chromosomes 1 to Z (Figure 3c). In conclusion, the RNA sequencing data showed that circular RNAs are highly expressed in DF1 cells.

### 3.4. Identification of Differentially Expressed Circular RNAs

**Figure 4:**
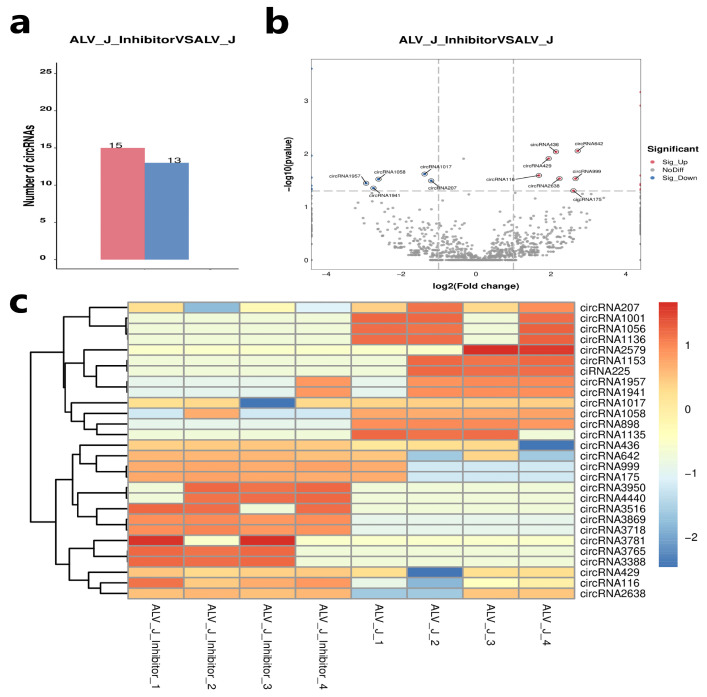
Identification of DE circular RNAs. (**a**) Statistics on the number of DE Circular RNAs in the ALV-J inhibitor and the ALV-J group DF1 cells. (**b**) Volcano map of DE Circular RNAs expressed in the ALV-J inhibitor and the ALV-J group DF1 cells. The red dots represent upregulated circular RNAs; the blue dots represent downregulated circular RNAs and the grey dots represent no difference. (**c**) Heat map of the DE Circular RNAs in the ALV-J inhibitor and the ALV-J group DF1 cells.

To further investigate the potential functions of these circular RNAs in ALV-J virus replication, we performed a differential expression analysis. A total of 28 circRNAs were identified as differentially expressed circRNA in DF1 cells of the ALV-J + 2-DG (ALV-J inhibitor) and the ALV-J group (Supplementary Table S3). Among them, 15 circRNAs were upregulated and 13 circRNAs were downregulated in the ALV-J + 2-DG group compared with the ALV-J group (Figure 4a,b). Among these differentially expressed circRNAs, eight were specifically expressed in the ALV-J + 2-DG group, and eight were specifically expressed in the ALV-J group (Supplementary Table S3). Heat maps showed that circRNA expression levels were roughly consistent in the same group (Figure 4c).

### 3.5. Validation of Differentially Expressed Circular RNAs

**Figure 5:**
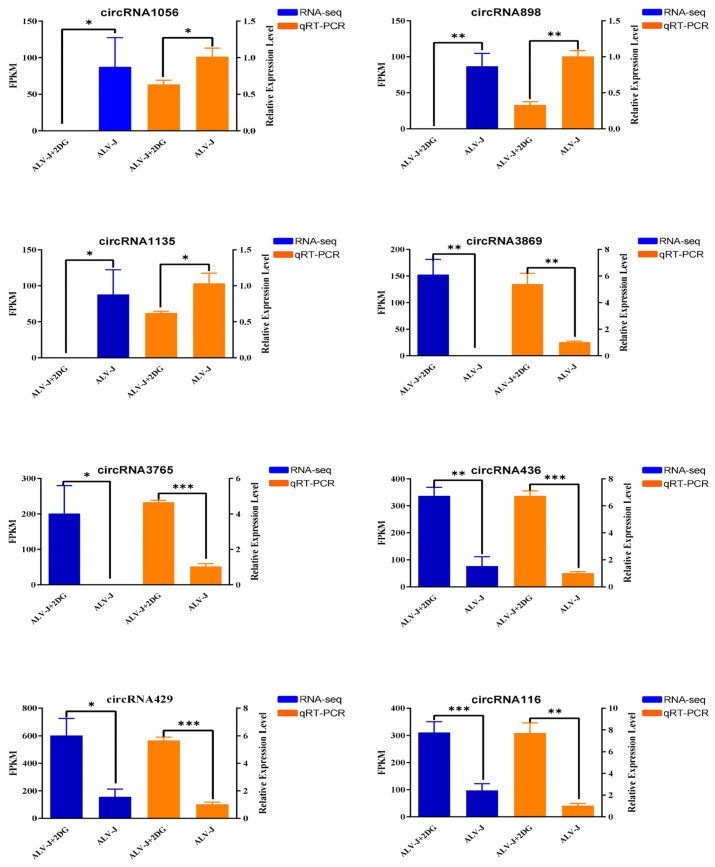
qRT-PCR validation of differentially expressed circular RNAs. Blue represents RNA-seq results and orange represents qRT-PCR results. *p* < 0.05 (*), *p* < 0.01 (**), or *p* < 0.001 (***) represent statistically significant, and all data are shown as means ± SD (standard deviation).

To validate the accuracy of RNA-seq data, we randomly selected eight DE circular RNAs for qRT-PCR analysis to verify whether their expression patterns were consistent with the sequencing data. The results of qRT-PCR show that the expression pattern of DE circRNAs was consistent with the RNA-seq results (Figure 5), which confirmed the reliability and repeatability of the results of the RNA-seq.

### 3.6. Functional Enrichment Analysis of DE circRNA Source Genes

**Figure 6:**
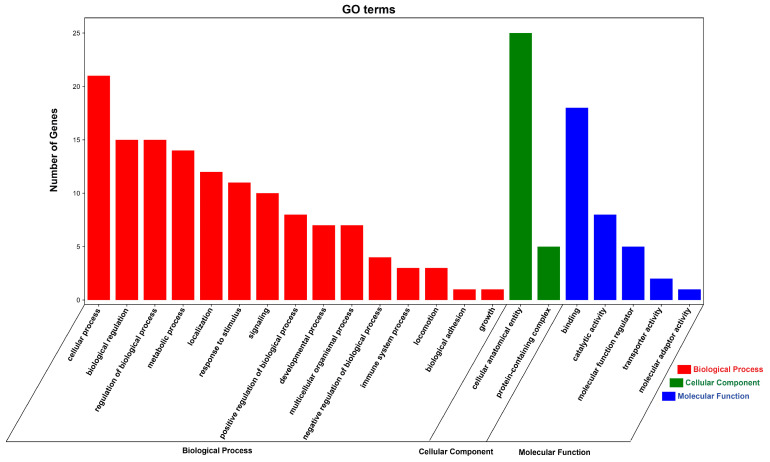
GO enrichment analysis of DE circRNA source genes. The red column represents the Biological Process, the green column represents the Cellular Component and the blue column represents the Molecular Function.

**Figure 7:**
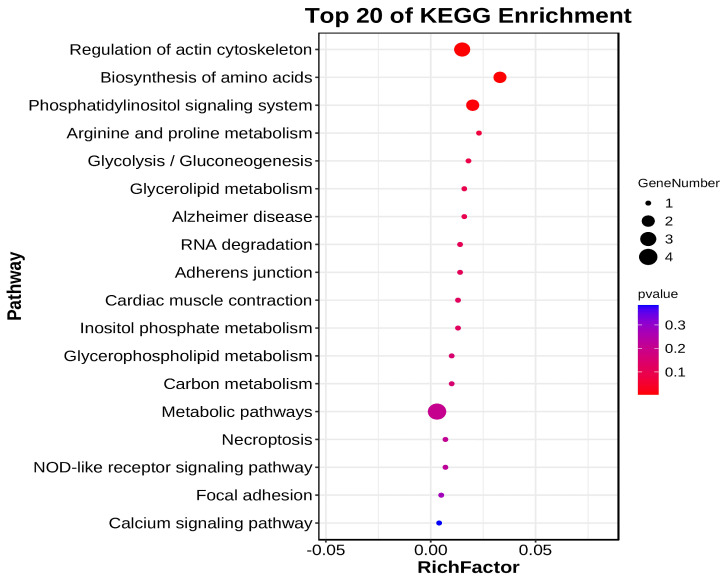
KEGG enrichment analysis of DE circRNA source genes. Top 20 pathway bubble diagram. Each bubble in Figure 7 represents a KEGG path, and the path name is shown in the legend on the left.

To further investigate the function of DE circRNAs, we performed GO and KEGG functional enrichment analysis on all DE circRNA source genes. The result of GO functional enrichment showed that these source genes were enriched in 23 GO terms (Supplementary Table S4). In all GO terms, DE CircrNA source genes were found to be enriched in biological process terms such as regulation of the biological process, response to stimulus, immune system process, biological regulation, and metabolic process (Figure 6, Supplementary Table S4). The result of KEGG enrichment showed that DE circRNAs Source Genes were enriched in 18 signaling pathways, including regulation of actin cytoskeleton, biosynthesis of amino acids, phosphatidylinositol signaling system, arginine and proline metabolism, glycolysis/gluconeogenesis, and glycerolipid metabolism. Among these pathways, DE circRNA source genes were especially enriched in glycolysis/gluconeogenesis and NOD-like receptor signaling pathways, which also indicates that our results are reliable (Figure 7, Supplementary Table S4).

### 3.7. Functional Enrichment Analysis of DE circRNA Target Genes

**Figure 8:**
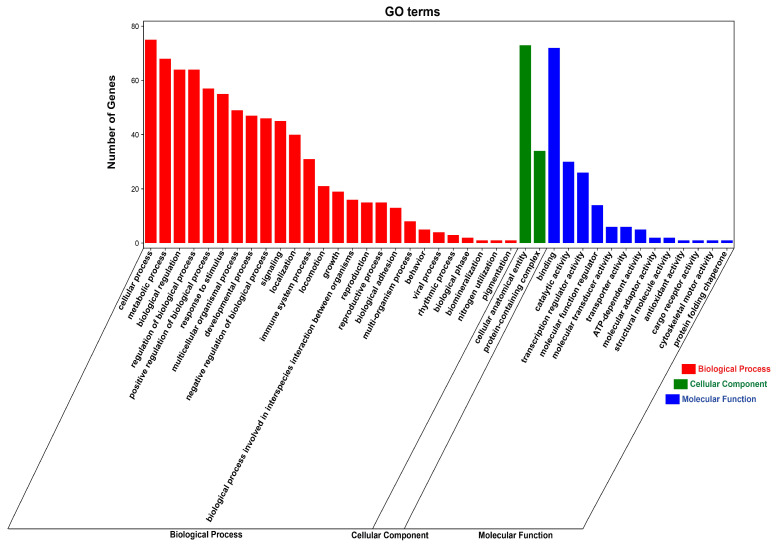
GO enrichment analysis of DE circRNA target genes. The red column represents the Biological Process, the green column represents the Cellular Component, and the blue column represents the Molecular Function.

**Figure 9:**
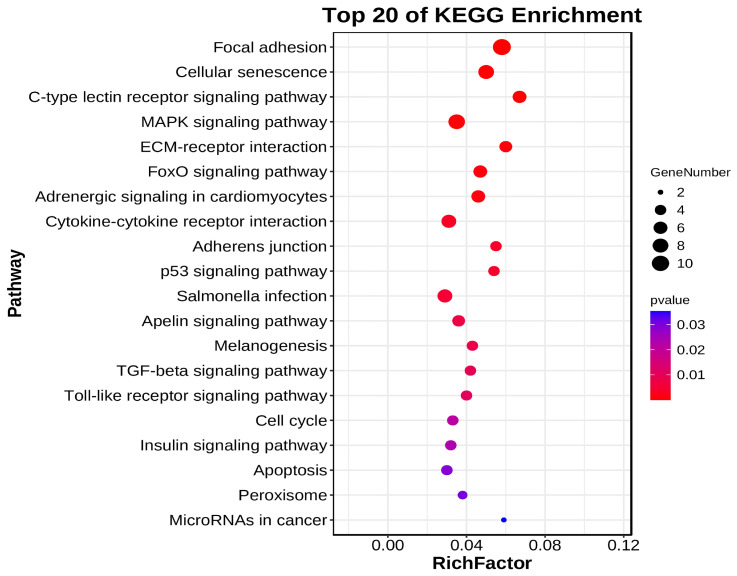
KEGG Enrichment Analysis of DE circRNA Target Genes. Top 20 pathway bubble diagram. Each bubble in Figure 7 represents a KEGG path, and the path name is shown in the legend on the left.

To further explore the function of DE circRNAs in regulating ALV-J virus replication, we used Targetscan (5.0) and miRanda software (3.3a) to predict circRNA–miRNA binding. The results showed that 1203 miRNAs may bind to 28 DE circRNAs (Supplementary Table S5). Based on the predicted results of miRNA that may interact with DE circRNA, we further used miRTarBase (6.1) and TarBase (9.0) databases to analyze the miRNA–target gene relationships associated with corresponding miRNAs. The results showed that 87 target genes might bind to these miRNAs (Supplementary Table S5). Based on the interaction analysis of circRNA and miRNA and the targeting relationship between miRNA and mRNA, we then constructed the circRNA–miRNA–mRNA relationship table to understand the ceRNA regulation mechanism of DE circRNAs (Supplementary Table S5). Additionally, we further performed GO and KEGG enrichment analyses to elucidate the potential role of circRNA target genes. The result of GO functional enrichment showed that these target genes were enriched in 41 GO terms (Supplementary Table S6). In all GO terms, DE CircrNA target genes were found to be enriched in positive/negative regulation of biological processes, immune system processes, metabolic processes, responses to stimuli, and viral processes (Figure 8, Supplementary Table S6). The result of KEGG enrichment showed that DE circRNAs target Genes were enriched in 88 signaling pathways, including MAPK signaling pathway, p53 signaling pathway, Toll-like receptor signaling pathway, Insulin signaling pathway, and Apoptosis, which were related to immunity or glycolysis (Figure 9, Supplementary Table S6).

## 4. Discussion

After the virus infects the host, it usually needs to use the host’s energy to maintain its normal replication, and glucose is the most important energy source in animals and is also the energy source for maintaining the virus replication [33]. The way of energy metabolism in normal cells is to transport glucose into the cell, and, after oxidative phosphorylation through the mitochondrial tricarboxylic acid cycle, a large amount of ATP is produced for energy supply. However, tumor cells are in a high metabolic state and need to consume a lot of energy and nutrients to maintain the ability of cell rapid proliferation, resulting in increased glucose intake by cells, enhanced glycolysis, and increased lactic acid. In the presence of sufficient oxygen, tumor cells also prefer glycolysis for energy metabolism, a phenomenon known as the “Warburg effect” [34]. The glycolysis process in vivo is divided into two stages, the first stage is the generation of pyruvate from glucose, and the second stage is the conversion of pyruvate into lactic acid. In this study, we found that ALV-J infection of DF1 cells resulted in increased glucose uptake, pyruvate, and lactic acid content, and significantly increased expression of glycolytic-related genes, which prompted us to think that ALV-J-infection was associated with DF1 cell glycolysis. The research found that NDV (Newcastle disease virus) elevates glucose uptake and glycolysis in infectious cells, and HBV (Hepatitis B virus) activates glycolysis to impede retinoic acid-inducible gene I (RIG-I)-induced interferon production [35,36]. These results were similar to our results. Studies have shown that the survival and pathogenesis of viruses that infect host cells depend on host cell glycolysis [8]. For example, glycolysis induced by SVA (Senecavirus A) is shown to facilitate virus replication by promoting lactate production [37]. Therefore, host glycolysis may affect the viral replication process. Then, we detected the expression of virus protein after inhibiting the glycolysis process of DF1 cells with 2-DG, and the results showed that 2-DG treatment could reduce the replication of the ALV-J virus, suggesting that inhibiting the glycolysis could reduce ALV-J virus replication in DF1 cells.

Our previous study found that 152 circRNAs were differentially expressed in normal chicken spleen tissue and ALV-J-infected chicken spleen tissue, suggesting that circRNA may play a role in the spleen tissue of ALV-J virus-infected chickens [38]. In the current study, we identified 11066 circRNA in the ALV-J + 2-DG group and the ALV-J Group DF1 cells, suggesting that circRNAs play an important role in the process of ALV-J virus infection. Most circRNAs are derived from exons, consistent with previous circRNA sequencing studies in chickens [39]. In addition, we screened 28 differentially expressed circRNAs based on transcriptome data, and we preliminarily understood the potential functions of circRNA in viral replication through the analysis of these differentially expressed circRNAs.

Studies have shown that circRNAs can influence gene or transcript expression through their source genes [40]. In this study, we found that 14 circRNA source genes were enriched in the biological processes of the metabolic process based on functional enrichment analysis of DE circRNA source genes. It has been reported that virus can perform their replication by altering host cell metabolism [41]. Among these 14 circRNA source genes, circRNA3388 (ALDH18A1) and circR-NA1056 (ENO1) may regulate ALV-J replication by affecting cell metabolism. Aldehyde Dehydrogenase 18 Family Member A1 (ALDH18A1) can participate in the regulation of the proline metabolism pathway in vivo, and then affect the survival and growth of cells [42], and Alpha-enolase (ENO1) plays an important role in the glycolytic processes and cell energy metabolism [43,44,45]. The investigation into the function of these source genes revealed that circRNAs play a vital role in virus infection and cell metabolism pathways. Through a pathway enrichment analysis of DE circRNA source genes based on the KEGG database, we found that these source genes were mainly enriched in some pathways, including biosynthesis of amino acids, arginine and proline metabolism, and glycolysis/gluconeogenesis. It has been reported that the African swine fever virus could promote its viral replication by regulating the host’s energy and amino acid metabolism, which indicates that the amino acids biosynthesis pathway had an important influence on viral replication [46]. Arginine and proline metabolism pathways also play an important role in the virus replication process, providing energy for its normal copy [42,47]. In addition, glycolysis and gluconeogenesis pathways were also reported to be associated with the virus infection [8,48], which indicated that glycolysis might influence the ALV-J replication process by regulating the expression of circRNAs.

Recent studies have shown that circRNAs could participate in cellular responses to Herpes Simplex Virus 1, human cytomegalovirus, and HIV infection through the circRNA–miRNA–gene regulatory axis, suggesting that circRNA and ceRNA mechanisms play an important role in viral pathogenesis and cellular immunity [21,22,23]. In the current study, we constructed a circRNA–miRNA–target gene regulatory axis for DE circRNAs to understand the potential role of DE circRNA target genes in ALV-J virus replication. Based on functional enrichment analysis of differentially expressed circRNA target genes, we found that 87 target genes were enriched in 41 GO terms, including the immune system process, metabolic process, viral process, and so on. RNA-seq reveals that DE circRNA source genes were enriched in the innate immune response after human umbilical vein endothelial cells (HUVECs) were treated with Hantavirus infection, which suggests that circRNAs may influence the viral infection process through host immune responses [49]. Similar to the functional enrichment analysis result of the DE cicrRNA source genes, some DE cicrRNA target genes were enriched in the metabolic process, which also indicated that cicrRNAs play a vital role in virus infection and cell metabolism pathways. Moreover, we found that some DE circRNA target genes were enriched in the viral process, including HSP90AB1, and IFIH1. It is reported that HSP90AB1 was a host factor that promotes porcine deltacoronavirus (PDCoV) replication and enhances transmissible gastroenteritis virus (TGEV) infection [50,51]. IFIH1 has also been found to restrict the replication of human respiratory syncytial virus and rhinovirus, and IFIH1 loss-of-function mutations could lead to severe viral respiratory infections in children [52]. The function of these target genes indicated that circRNAs play an important role in the virus infection process. Based on the results of the pathway enrichment analysis of DE circRNA target genes, we found that some target genes were enriched in the insulin signaling pathway. It has been reported that defective insulin signaling results in enhanced glycolysis affecting the responses to pathogens [53]. In addition, it was found that the loss of PFK-2 content could reduce insulin signaling, elevate the levels of early glycolytic intermediates, and impair dynamically regulated glycolysis ability [54]. These results suggest that glycolysis may act on these signaling pathways by regulating the expression of DE circRNA target genes, ultimately affecting the replication of the ALV-J virus.

## 5. Conclusions

In this study, we investigated the effect of glycolysis on the replication of the ALV-J virus in DF1 cells. We found that glycolysis could inhibit the replication of the ALV-J virus in DF1 cells. Moreover, we identified 28 circRNAs that were differentially expressed between ALV-J-infected and ALV-J-infected cells treated with glycolysis inhibition according to RNA-seq data. Based on the results of the function enrichment analysis of DE circRNA source and target genes, we speculated that glycolysis might affect the replication of the ALV-J virus in DF1 cells through signaling pathways such as glycolysis/gluconeogenesis, the MAPK signaling pathway, NOD-like receptor signaling pathway, p53 signaling pathway, and Insulin signaling pathway. Our study reveals the effect and the potential regulatory mechanisms of glycolysis on the replication of the ALV-J virus, broadening the insight into the role of glycolysis in the transmission of chicken ALV-J virus.

## Figures and Tables

**Table 1:** Primers used for qRT-PCR.

Gene	Primer Sequence (5′-3′)	Product Size (bp)	Annealing Temperature (°C)
PKM2	F: GGCACCCACGAGTATCAT	205	60
	R: CATTGTCCAGCGTCACTTT		
HK1	F: CCTCTTGGCTTCACATTC	231	60
	R: TTCACAGTTTGGGTCTTCAT		
HIF1A	F: TTGACAAGGCATCCATTA	157	60
	R: TCCTCAGAAAGCACCATA		
LDHA	F: TGGGCATCCATCCTCTGA	168	60
	R: CCTGCTTGTGAACCTCCT		
GLUT1	F: TGTTTGGCTTGGACTTGAT	171	60
	R: TCTTGAGGACGCTCTTGG		
PFKP	F: GCCACAACAAACCTATAACA	241	60
	R: ATCAAAGGCAGACGAACA		
β-actin	F: GAACCCCAAAGCCAACAGAGAG	142	60
	R: ATCACCAGAGTCCATCACAATACCA		

**Table 2:** Primers for DE circRNAs.

circRNAs	Source Gene	Primer Sequence (5′-3′)	Product Size (bp)
CircRNA116	SPPL3	F: TGCTTTCCTTTTGCTCCCGA	199
		R: GACTGGAATCCACGAGGGAA	
CircRNA429	MICAL2	F: ACCCCCACTAGAAATCCTTGC	195
		R: CGTAGGACAGCAAATTGCCC	
CircRNA436	DENND5A	F: CCAGAGGCCAAGCACCAATG	185
		R: GGCTGGCACCATCTCGG	
CircRNA898	C2CD3	F: CAGGGACCTGCTGAGAAGAC	166
		R: GATCAGCTGAGCCCATTGCC	
CircRNA1056	ENO1	F: CCTTGTCAGAGTAGCCAGCC	136
		R: CTGGGGTGTGATGGTGAGTC	
CircRNA1135	SLC9A6	F: GAGCACGAAGGCAGAAAGCG	194
		R: GCAAATGCCATGGCTCCTCG	
CircRNA3765	FAF1	F: CCCATCGAGAAGTCCAGCGG	155
		R: TGTGGGGCCATATGCTGGTC	
CircRNA3869	DOCK1	F: GGAGTCTTTGCTGCAGCTCT	162
		R: GCATCAAACACCAACGTGTCA	
β-actin	/	F: GAACCCCAAAGCCAACAGAGAG	142
		R: ATCACCAGAGTCCATCACAATACCA	

## Data Availability

Data presented in this study are available upon request from the corresponding author.

## Supplementary Materials

The following supporting information can be downloaded at: https://www.mdpi.com/article/10.3390/ani14172500/s1, Table S1: Mapping statistic; Table S2: Statistical of annotated circRNAs; Table S3: Statistical of differential circRNAs; Table S4: Enrichment analysis of source genes; Table S5: Bioinformatic analysis of circRNA-miRNA-mRNA; Table S6: Enrichment analysis of target genes.
